# Ethnoveterinary plants of Ankober District, North Shewa Zone, Amhara Region, Ethiopia

**DOI:** 10.1186/1746-4269-10-21

**Published:** 2014-02-11

**Authors:** Ermias Lulekal, Zemede Asfaw, Ensermu Kelbessa, Patrick Van Damme

**Affiliations:** 1 Laboratory for Tropical and Subtropical Agriculture and Ethnobotany, Department of Plant Production, Faculty of Bio-Science Engineering, Ghent University, Coupure links 653, 9000 Ghent, Belgium; 2 Department of Plant Biology and Biodiversity Management, College of Natural sciences, Addis Ababa University, P.O. Box 3434, Addis Ababa, Ethiopia; 3 Department of Crop Science and Agroforestry, Faculty of Tropical Agrisciences, Czech University of Life Sciences Prague, Kamycka 129, 165 21 Prague 6-Suchdol, Czech Republic

**Keywords:** Ethnomedicine, Fidelity level, Traditional knowledge, Informants consensus factor, Preference ranking, Traditional healers

## Abstract

**Background:**

Traditional herbal preparations for addressing veterinary problems have been applied in Ankober District, Ethiopia, for generations. However, the millennia-old ethnoveterinary knowledge of the community, and the plants are subjected to loss without being scientifically documented due to anthropogenic and environmental threats. Hence, this study aims at providing a comprehensive documentation on ethnoveterinary plant knowledge of the people in order to preserve the fast-eroding knowledge and resources of the area.

**Methods:**

Semi-structured interviews, focus group discussions, participant observation and walk-in-the-woods methods were used to gather ethnoveterinary data. Informant Consensus Factor (ICF) and Fidelity level (FL) values were calculated using quantitative approaches so as to check the level of informants' agreement on plant use and healing potential of ethnoveterinary medicinal plant species, respectively. Indigenous knowledge on use of medicinal plants for ethnoveterinary purposes among different informant groups was compared using One-way ANOVA and t-tests.

**Results:**

A total of 51 plant species representing 50 genera and 35 botanical families used in the treatment of 33 different ailments were identified. Medicinal plant species belonging to families Asteraceae, Asclepiadaceae, Euphorbiaceae and Ranunculaceae were reported to be of frequent use in the local ethnoveterinary medical system. Roots (65%, 33 species) were most often utilized for remedy preparation. Highest ICF values were recorded for gastro-intestinal (0.71) ailments depicting best agreement on knowledge of medicinal plants used to treat aliments in this category. *Embelia schimperi* Vatke showed highest fidelity level value (90%) to treat gastro-intestinal diseases showing conformity of knowledge on this species' healing potential. Significant difference (P<0.05) was observed in average number of therapeutic plants reported by senior members of the community than younger groups. *Embelia schimperi Vatke* and *Rubus steudnerii* Schweinf. were the most-preferred species to treat diarrhoea.

**Conclusion:**

The study indicated that indigenous knowledge on ethnoveterinary medicinal plant use is still rich and active in the District. Species with recorded highest consensus for curative role are a useful pool for further phytochemical and pharmacological validation for better utilization. Declining wild medicinal flora of the area calls for implementation of a coordinated complementary *in situ* and *ex situ* conservation strategy.

## Background

Livestock husbandry is a mainstay in the livelihood of more than 70% of Ethiopians [1]. Since the country’s economy is mainly based on agriculture, the livestock subsector is positively important in that it contributes about 16% of the national GDP and 45% of agricultural GDP [2]. About 90% of the crop production in Ethiopia depends on animal draft power [3]. Livestock also offers the only way of survival in many harsh environments on top of serving as a driving force of food security in the country [4]. Ethiopia has the highest number of livestock in Africa which is estimated to 53.3 million heads of cattle, 25.5 million sheep, 22.7 million goats, 5.7 million donkeys, 2 million horses, 1.1 million camels and 49.2 million chicken; hence the country is listed among the top 10 countries in the continent known for their livestock wealth [5]. Despite this large number of livestock and its important economic potential, the sector has not developed beyond a subsistence type of venture, whereas it also remained with low outputs for different reasons of which animal diseases are among the top factors [6].

Diseases are the prime causes of poor livestock productivity in many developing countries [7]. According to Admassu [8], the aggregate annual economic loss in Ethiopia from livestock diseases (through direct mortality, and reduced productive and reproductive performance) was estimated at US$ 150 million. Fromsa and Jobre [9] estimated annual economic loss from bovine hydatidosis to be at US$ 101 million in the country. Although disease-free livestock products (for in-house consumption and export purposes) are mandatory to ensure consumer’s health and to reap more earnings from the sector, it is hardly possible to provide an overall conventional veterinary medical services in Ethiopia and most developing countries, hence the sector remained with low output [4]. According to Duguma [6], inadequate veterinary health professionals, scarce and erratic supply of veterinary drugs, high cost of equipment and drugs, absence of government-based livestock health policies, presence of counter-productive livestock health policies and poor infrastructure are mentioned as some of the major factors that made livestock raisers in developing countries to rely more on ethnoveterinary medicine than the modern medical system.

Settled and nomadic livestock raisers from many countries have developed their own indigenous knowledge on ethnoveterinary practices through age-old cultural contact with curative plants, trial and error experiments and empirical observations to treat various livestock ailments [6]. Traditional knowledge on use of medicinal plants, however, is subjected to loss in the absence of incessant cultural interaction with medicinal species [10,11]. Reliance on ethnoveterinary knowledge in different countries is mostly reinforced by the strong dependence on livestock for livelihood and the richness in cultural history, ancestral knowledge and biodiversity [12]. According to Cetinkaya [13], demographic, economic, socio-political, ecological, religious and cultural factors existing in a community are key drivers shaping traditional knowledge in a given society.

Millennia-old traditional knowledge on ethnoveterinary folk medicine has been recognised during the 32nd session of UNESCO as one of the important components of indigenous cultural heritage that need to be studied, sustained and protected [14]. Since then, scientific studies and documentation of indigenous knowledge on ethnoveterinary medicinal plants have been initiated in many countries; to mention but few: Kenya [15], Mediterranean areas of Albania, Italy, Morocco, Spain, Egypt, Greece, Algeria and Cyprus [16], Canada [17], South Africa [18], Pakistan [19], Uganda [12], Brazil [20], Argentina [21], India [22], Nigeria [23] and Spain [24] and Germany [25]. However, the effort is still quite insignificant when compared to the undocumented global ethnoveterinary plant lore.

In Ethiopia, ethnoveterinary practices were reported to be the only options to cure livestock ailments till the advent of modern veterinary services which were started as late as 1908 [8]. Although a gain from the richest livestock wealth of the country is directly related to safeguarding livestock health, conventional veterinary medical system is yet very poor in the country [26]. Abebe [27], stated that traditional plant remedies are major sources of therapeutics for nearly 90% of the livestock population in Ethiopia. Moreover, about 95% of all forms of traditional medicinal preparations in the country are also reported to be of plant origin [28]. Thus, the deep-seated custom of using medicinal plants led Ethiopian cultural communities to know medicinal properties of many plants that are used to treat livestock ailments [29]. However, the rich indigenous knowledge on many of the traditional plant remedies is subjected to loss as it has mainly been passed orally for generations without being properly nor scientifically documented [30].

Despite the significant role played by ethnoveterinary plants for treating livestock ailments in both settled and pastoralist areas of Ethiopia, a very limited attempt has been done to explore, document and promote these widely used ethnoveterinary plants in the country. Moreover, Ethiopian medicinal plants and the associated indigenous knowledge are continuously threatened due to factors like deforestation, overexploitation, overgrazing, habitat loss and degradation, agricultural land expansion and acculturation [31]. Hence, it is a timely endeavour to document, promote and conserve the country’s ethnoveterinary medicinal plant lore. Such documents are important to define and maintain cultural identity of the people [13] in addition to serving as keys towards establishing people-centred natural resource management systems [32], and potentials for scientific discovery of new lead compounds used in the development of modern drugs [33].

Although Ethiopian ethnoveterinary medicinal inventories including those of [7,34-44], have attempted to document veterinary importance of traditional medicinal plants in some cultural groups, it is found insignificant when compared to the 85 different ethnolinguistic communities found in the country, which have remained largely unexplored. Hence, the present research aims to fill this gap by documenting the wealth of indigenous knowledge on utilization, management and conservation of ethnoveterinary plants used in Ankober District, north Shewa Zone, Ethiopia, which has never been explored for its ethnoveterinary wealth. It also aims to identify and document marketable medicinal plants of the District so as to identify the economic potential of medicinal plants in the area. In addition, it aims to select candidate ethnoveterinary plant species with high informants’ consensus and fidelity level values for further phytochemical and pharmacological analyses in subsequent studies.

## Methods

### Study area and ethnographic background

**Figure 1:**
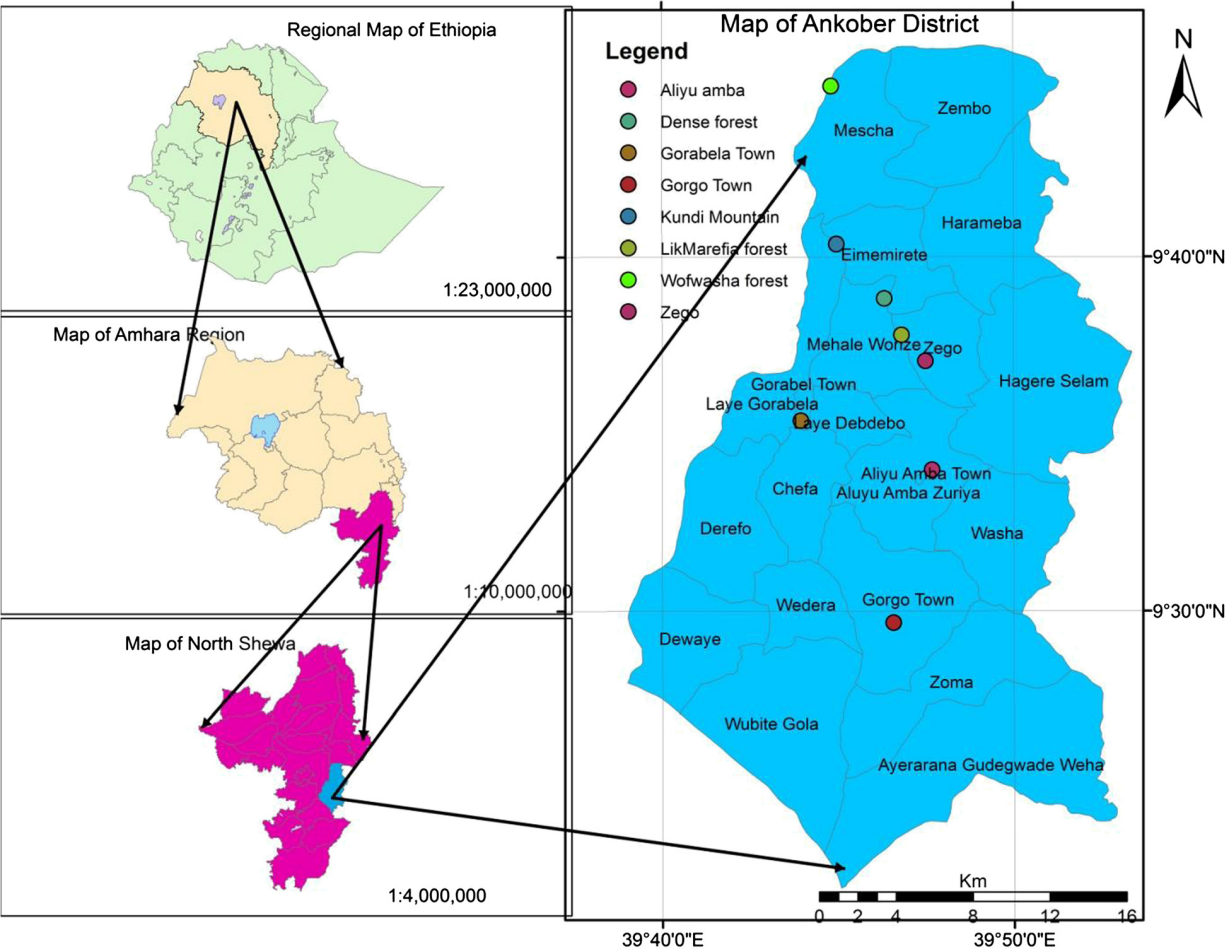
**Map of the study area (*developed using ArcGIS 10*).**

This study was conducted in Ankober Distrcit, located at 9° 22’ 0”- 9° 45’ 0” N and 039° 40’ 0”- 039° 53’ 0” E in north Shewa Zone of Amhara National Regional State, north-central Ethiopia (Figure 1). The District is perched on the eastern escarpment of the Ethiopian highlands and located at 172 km north of Addis Ababa, the Ethiopian capital, and 42 km to the east of Debre Berhan town (the north Shewa Zone capital). Ankober District is bordered in the north by Tarmaber District, south by Asagirt District and west by Basonaworana District of Amhara Region. The eastern part shares its border with Gachine Special District of the Afar Region [45]. Elevation in Ankober District ranges from 1300 m asl near Addis Alem area to 3700 m asl at Kundi Mountain. Annual rainfall in the District ranges 1000 to 1400 mm and cold temperature is prominent for most of the year [46]. The main administrative centre of the District is located at Gorabela/Ankober town that has historical significance as it has been the seat of the Ethiopian emperors from 1270 for centuries [45].

The indigenous people inhabiting the area belong to the Amhara ethnic group. They speak Amharic language, the national language of Ethiopia. The District has a total population of 83,260 (42,180 men and 41,080 women) of whom only 6,272 (7.5%) are urban inhabitants [5]. Ankober has a population density of 113.72 individuals/km^2^, which is slightly less than the north Shewa Zone average of 115.3 persons/km^2^[46]. About 92.52% of the people in Ankober belong to the Ethiopian Orthodox Tewahdo Christianity and 7.41% are Muslims.

Ankober District homes Dense and Likmarefia forests and part of the Wof Washa natural forest. These forests are rich in biodiversity and harbour economically important tree species including *Hagenia abyssinica* (Bruce) J.F. Gmel., *Olea europaea* L. subsp. *cuspidata* (Wall. ex G. Don), *Juniperus procera* L*.*, *Podocarpus falcatus* (Thunb.) Mirb. and *Nuxia congesta* R. Br. ex Fresen [47]. They are also home to very diverse wildlife and bird species including the IUCN red-listed endemic bird species *Serinus ankoberensis*[48].

### Livestock population and status of veterinary services in the District

The livestock population in the District was estimated to 61,499 heads of cattle, 33,265 sheep, 54,493 goats, 8,802 donkeys, 395 horses, 244 camels, 221 mules and 47,756 chicken in the year 2012 [46]. At the same time, there were only seven rural veterinary clinics in the District located at Mescha, Haramba, Gorgo, Aliyu Amba, Wedera, Derefo and Lay Gorebela kebeles (Lowest administrative units in Ethiopia). The remaining 15 kebeles did not have veterinary clinics at all. There were only eight veterinarians (1 DVM, 2 Bsc and 2 Diploma holders with 3 assistants) working in the District in 2012 [46]. This was found insufficient to provide proper health services for a total of more than 200,000 heads present in the District [46]. Blackleg, pasteurellosis, Newcastle disease, lumpy skin disease, foot and mouth disease, African horse sickness and various parasitic diseases are reported as the most common disease types affecting the District [46]. The local people in Ankober have long been dependent on traditional medicinal plants accessed from natural forest patches, cultivated land and field margins to manage various animal health problems.

### Selection of informants

Representative informants and knowledgeable traditional medicine practitioners of the District were selected using systematic random and purposive sampling approaches in the manner described by Martin [49]. The total number of informants involved in the ethnomedicinal survey was 352 (235 men and 117 women). Informants’ ages ranged 20– 89years (122 were between 20–39 whereas 230 were ≥ 40years old). All twenty-two kebeles of Ankober District were included in this investigation. Peer-recommendations from community members, elderly people and knowledgeable inhabitants helped in nominating 88 traditional herbalists (68 men and 20 women) who participated as key respondents following Davis and Wagner [50], whereas general informants were sampled during random visits made to houses in the study kebeles. Informed consent was obtained from each informant who participated in this study after explaining the purpose of the study and assuring them of the most responsible judicial use in the resulting information before the start of interviews.

### Ethnobotanical data collection

Ethnoveterinary data were collected during six different field visits conducted between June 2009 and May 2011. Data were collected in different seasons over the different years with the objective of including all kebeles in the District and collecting plant specimens during the respective flowering seasons. Market survey and checking of reliability of informants’ medicinal plant use knowledge were conducted between 29 December 2012 and 9 February 2013.

Semi-structured interviews, focus group discussions, participant observation and walk-in-the-woods were used to collect ethnoveterinary data as described by Martin [49], Cotton [51]. Informants were interviewed individually in the local Amharic language. Semi-structured interviews addressed questions regarding name, age, gender, level of education, occupation, religion and ethnic background of each informant. The individual semi-structured interviews included local names of medicinal plants, ailments treated, habitat of the species, distance from the house to gathering sites, seasonality of species, marketability of species, degree of management (wild/cultivated), abundance, parts used, condition of plant part used (fresh/dried), other ingredients or additives (if any), methods of remedy preparation, remedy preservation (storage), dosage prescriptions, routes of remedy administration, noticeable adverse effects of remedies, use of antidotes for adverse effects, taboos/beliefs related to collection and use of plants, source of knowledge, method of indigenous knowledge transfer, other uses of species mentioned, existing threats and traditional conservation practices (if any), and in the case of traditional healers number of years of service as healer and income earned per animal treated for an ailment following the methods used by previous researchers [49,51-53].

All semi-structured interviews were followed by independent walk-in-the-woods activities, which gave an opportunity for more discussion with individual informants and the practical identification and collection of traditionally used medicinal plants in their natural environment. This method was combined with participant observation through which reliability of the information collected was ascertained on collection methods and preparation of specific remedial parts as described by Cotton [51], Alexiades [52]. In addition, focus group (one focus group per kebele with average number of 5 participants) discussions were undertaken to gain further information on medicinal plants knowledge of the community and prove the reliability of the data collected through semi-structured interviews [49].

Preference ranking was conducted using fifteen key informants (ten men and five women) in the manner recommended by Martin [49] to identify the-most preferred species used for treating the most commonly reported gastro-intestinal disease in the area. Major markets of Ankober District i.e., Gorebella, Aliyuamba, Gorgo, Haramba, Derefo, and Zego were surveyed for availability, price and unit of measurement of marketable medicinal plants (if any).

Voucher specimens of medicinal plants were collected on the field with the help of traditional healers and local field assistants. Specimens were dried, numbered, labelled, pressed, identified and deposited at the National Herbarium (ETH) in Addis Ababa University. Identification of specimens was performed both in the field and later at ETH using taxonomic keys and floras [54-61] and by comparison with authenticated herbarium specimens.

### Data analysis

Ethnoveterinary data on medicinal plants used in Ankober District were entered in an excel spreadsheet (Microsoft corporation, 2007) and organised for statistical analysis. Descriptive statistics was applied to compute the number and percentage of species, genera and families of ethnoveterinary medicinal plants, their growth forms, proportions of parts harvested, modes of remedy preparation and routes of administration. Preferences of ethnoveterinary plant species used to treat the commonly reported livestock ailments in the study area were ranked by adding the values/scores of preferences given by respective informants so as to identify the most-preferred medicinal plant species to treat the most frequently reported disease type in the area following the relevant standard methods [49,52].

Informant Consensus Factor (ICF) values [62] were calculated to determine the most important livestock ailment categories in the District and identify potentially effective medicinal plant species in respective disease categories. Accordingly, reported traditional remedies and corresponding livestock ailments occurring in the District were categorized into eight disease categories and the ICF values were obtained by computing number of use citations in each disease category (n_ur_) minus the number of times species used (n_t_), divided by the number of use citations in each category minus one.

$$\mathrm{ICF}=n_{\mathrm{ur}‒}n_{t/}n_{\mathrm{ur}‒1}$$

An index of Fidelity Level (FL) given by FL=Ip/Iu X 100 [52], where Ip is the number of informants who independently indicated the use of a species for treating a particular disease, and Iu the total number of informants who reported the plant for any given disease was used to determine the relative healing potential of reported ethnoveterinary plant.

Traditional knowledge dynamics on use of medicinal plants by men and women, young to middle aged (23–39 years) and elderly (40–89 years); literate (completed at least primary education) and illiterate; knowledgeable (key) and local (encountered randomly) informants was compared using *t*-test and one way ANOVA at 95% confidence level between means, since data are normally distributed, using KyPlot 5.0 software.

## Results

### Ethnoveterinary medicinal plant diversity in Ankober District

A total of 51 ethnoveterinary medicinal plant species representing 50 genera and 35 botanical families were identified in the District (Table 1). Thirty-one percent of the botanical families were represented by more than one species. The highest number of species was recorded for family Asteraceae (4 species, 8%) followed by Asclepiadaceae, Euphorbiaceae and Ranunculaceae (3, 6% each). Seven of the reported botanical families i.e., Fabaceae, Lamiaceae, Menispermaceae, Myrsinaceae, Plantaginaceae, Rubiaceae and Solanaceae were represented by 2 (4%) species each. The remaining 24 (69%) families had a single species representation. About 6% (3 species) of the medicinal plant species are endemic to Ethiopia (Table 1).

**Table 1:** List of ethnoveterinary medicinal plants used for treatment of livestock ailments: scientific name; family; local name; growth form; ailment treated; plant parts used; condition of plant part uses; methods of preparation and application, route of administration, plant part mixed with and voucher number

**Scientific name**	**Family**	**Local (Amharic) name**	**Growth form**	**Ailment treated**	**Part used**	**CPU**	**MP AP**	**RA**	**Part mixed with**	**Voucher no ermiasLX**
*Achyranthes aspera* L.	Amaranthaceae	Telenj	H	Nasal infection	R	F	5	Na		104
				Ophthalmic infection	L	F	4	Op		
				Minor bleeding	L	F	3	De		
*Aeonium leucoblepharum* Webb ex A. Richard	Crassulaceae	Yefeyel Dabo	H	Sore	L	F	2	De		885
				Retained placenta/fetal membrane	L	F	5	O		
*Allium sativum* L.	Alliaceae	Nech Shinkurt	H	Blackleg	BU	F/D	5	O	8, 11	828
				Dermatophilosis	BU	F/D	2	De		
				Mange	BU	F/D	2	De		
				Scabies	BU	F/D	2	De		
				Ringworm	BU	F/D	2	De		
				Parasitic leech	BU	F/D	5	Na		
				Lice infestation in chicken	BU	F	4	De		
				Helminthiasis	L	F	5	O		
*Apodytes dimidiata* E. Mey. ex Arn	Icacinaceae	Yetemenja Inchet/Donga	T	Rabies	SB	F	5	O	5	887
				African horse sickness	SB	F	5	O		
*Asparagus africanus* Lam.	Asparagaceae	Seritie	S	Coccidiosis	R	F	1	O	7	189
*Calotropis procera* (Ait.) Aitf	Asclepiadaceae	Kimbo	H	Mange	R	F	4	De		894
*Calpurnia aurea* (Ait.) Benth.	Fabaceae	Digita	S	Tick infestation	L	F	4	De		76
				Helminthiasis	L	F	5	O		
				Snake bite	R	F	5	O	3, 11	
				Sore	L	F/D	3	De		
				Parasitic leech	BU	F/D	5	Na		
*Carissa spinarum* L.	Apocynaceae	Agam	S	Helminthiasis	R	F	4	O		16
				Parasitic leech	L	F	4	De		
*Cissampelos mucronata* A. Rich.	Menispermaceae	Ingochit hareg	C	Cowdriosis	P	F	5	O	14	888
				CBPP	P	F	5	O	40	
*Clematis hirsuta* Perr. & Guill.	Ranunculaceae	Azo hareg	C	Blackleg	R	F	5	O	3, 8	18
				Pleuropneumoniasis	L	F	5	O		
*Clutia abyssinica* Jaub. & Spach	Euphorbiaceae	Feyele Fej	S	Bloody diarrhoea	L	F	5	O		10
				Nasal infection	R	F	5	O		
				Ophthalmic infection	L	F	4	O		
				Mucal diarrhoea	R	F	5	O		
				Bloat	L	F	5	O		
*Croton macrostachyus* Del.	Euphorbiaceae	Bisana	T	Ringworm	L	F	4	O		17
				Dermatophilosis	L	F	4	O		
				Mange	L	F	4	O		
				Scabies	L	F	4	O		
				Wound	L	F	4	O		
				Minor bleeding	L	F	3	O		
				Sore	L	F/D	3	O		
*Cucumis ficifolius* A. Rich.	Cucurbitaceae	Yemdr Imbuay	H	Coccidiosis	Fr, R	F/D	1	O		68
				Cowdriosis	R	F	5	O	3	
				Hepatitis	Fr	F/D	5	O		
				Wound	L&R	F	3	O		
*Debregeasia saeneb* (Forssk.) Hepper & Wood	Urticaceae	Wenz admek	S	Mange	R	F	2	O		890
				Lumpy skin disease	R	F	2	O		
				Scabies	R	F	2	O		
*Discopodium penninervium* Hochst.	Solanaceae	Ameraro	S	Blackleg	L	F	5	O		606
				Wound	L	F	2	O		
				Minor bleeding	L	F	3	O		
*Dodonaea angustifolia* L. f.	Sapindaceae	Kitkita	S	Bloat	RB	F/D	5	O		20
				Sudden diarrhoea	R	F	5	O		
				Ringworm	R	F	2	O		
				Scabies	R	F	2	O		
*Dracaena afromontana* Mildbr.	Dracenaceae	Merko	S	Blackleg	R	F	5	O		892
				Scabies	R	F	2	O		
				Dermatophilosis	R	F	4	O		
*Dregea schimperi* (Decne.) Bullock	Asclepiadaceae	Yedikula Kend	C	Snake bite	R	F	5	O		798
				Wound	L	F	4	O		
*Embelia schimperi* Vatke	Myrsinaceae	Inkoko	C	Bloat	Fr	F/D	5	O		505
				Mucal diarrhoea	Fr	F	5	O		
				Bloody diarrhoea	Fr	F/D	5	O	4	
*Gomphocarpus fruticosus* (L.) Ait. F.	Asclepiadaceae	Ashkla Hareg	H	Bloat	P	F	5	O	51	133
				Poor appetite	R	F	5	O		
				Sudden diarrhoea	Fr, R	F/D	5	O		
*Hypericum quartinianum* A. Rich.	Hypericaceae	Ameja	S	Snake bite	R	F	5	O		889
				Minor bleeding	R	F	4	O		
*Hypoestes aristata* (Vahl.) Soland.	Acanthaceae	Telenj	H	Wound	L	F	3	O		599
				Minor bleeding	L	F	3	O		
*Inula confertiflora* A. Rich.**	Asteraceae	Woynagift	S	Lumpy skin disease	L	F	5	O		877
				Mange	R	F	4	O		
				Ringworm	R	F	4	O		
*Juniperus procera* L.	Cupressaceae	Yeabesha Tsid	T	Retained placenta/fetal membrane	R	F	5	O		874
*Leonotis ocymifolia* (Burm.f.) Iwarsson	Lamiaceae	Ras Kimir	S	Anthrax	R	F	5	O	26, 27	871
				Blackleg	L	F	5	O		
*Maesa lanceolata* Forssk.	Myrsinaceae	Kelewa	S	Mange	Fr	F	4	O		42
				Tick infestation	L	F	4	O		
				Dermatophilosis	Fr	F	2	O		
				Helminthiasis	Fr	F	5	O		
				Parasitic Leech	Fr	F	5	O		
*Nuxia congesta* R. Br. ex Fresen.	Loganiaceae	Askwar	T	Calf pneumonia	L	F	5	O		897
				Cowdriosis	SB	F	5	O		
				CBPP	SB	F	5	O	9	
*Ocimum lamiifolium* Hochst.	Lamiaceae	Dama Kessie	S	Bloat	L	F	5	O		864
				Mucal diarrhoea	L	F	5	O		
				Poor appetite	L	F	5	O		
				Bloody diarrhoea	L	F	5	O		
*Olea europaea* L. subsp. cuspidata (Wall. ex G.Don)	Oleaceae	Woyra	T	Mange	St	F	4	O	21, 41	19
				Ringworm	R	F	2	O		
				Lumpy skin disease	R	F	2	O		
*Pavetta abyssinica* Fresen.	Rubiaceae	Seged achawach	S	Blackleg	SB	F	5	O		485
				Broken limb in calf	SB	F	3	O		
*Pentas lanceolata (Forssk.) Defiers*.	Rubiaceae	Yejib Mirkuz	S	Anthrax	R	F	5	O	30	213
*Phytolacca dodecandra* L' Herit.	Phytolaccaceae	Indod	S	Parasitic leech	L	F	5	O		243
				Mange	R	F	2	O		
				Helminthiasis	R	F	5	O		
				Lice infestation in chicken	L	F	4	O		
				Lumpy skin disease	R	F	2	O		
*Plantago lanceolata* L.	Plantaginaceae	Wusha milas tinishu	H	Rabies	R	F	5	O		860
*Plantago palmata* Hook.f.	Plantaginaceae	Wusha milas tiliku	H	Rabies	R	F	5	O		895
*Podocarpus falcatus* (Thunb.) Mirb.	Podocarpaceae	Zigba	T	Ringworm	SB	F	2	O		197
				Mange	SB	F	2	O		
*Polyscias fulva* (Hiern) Harms	Araliaceae	Yezingero wonber	T	Minor bleeding	L	F	2	O		823
*Ranunculus multifidus* Forssk.	Ranunculaceae		H	Dermatophilosis	R	F	4	O		858
*Ricinus communis* L.	Euphorbiaceae	Gulo	S	Mange	Fr	F	4	O	21,33	33
				Scabies	Fr	F	4	O		
				Ringworm	Fr	F	4	O		
				Retained placenta/fetal membrane	R	F	5	O		
*Rubus steudnerii* Schweinf.	Rosaceae	Amoch	S	Bloat	LT	F	5	O		242
				Mucal diarrhoea	R	F	5	O		
				Bloody diarrhoea	R	F	5	O	32	
				Blackleg	R	F/D	5	O		
Senecio myriocephalus Sch. Bip. exA. Rich.**	Asteraceae	Sibut	S	Pasteurellosis	R	F	5	O		891
*Sida schimperiana* Hochst. ex A. Rich.	Malvaceae	Garda	S	African horse sickness	R	F	5	O		153
*Smilax aspera* L.	Smilacaceae	Ashkla hareg	C	Snake bite	L	F	5	O		853
*Solanecio gigas* (Vatke) C. Jeffrey**	Asteraceae	Dengorita	T	Hepatitis	L	F	5	O		531
*Stephania abyssinica* (Dill. & A. Rich.) Walp.	Menispermaceae	Yeait hareg	H	CBPP	L	F	5	O		130
				Calf pneumonia	L	F	5	O		
*Tephrosia interrupta* Hochst. & Steud ex Engl.	Fabaceae	Gerengere	S	Coccidiosis	R	F	1	O	30	893
*Thalictrum rhynchocarpum* Dill. & Rich.	Ranunculaceae	Sire bizu	H	African horse sickness	R	F	5	O		535
*Trichocladus ellipticus* Eckl. & Zeyh.	Hamamelidaceae	Abil wuha	T	Ophthalmic infection	L	F	4	O		896
				African horse sickness	R	F	5	O		
*Triumfetta brachyceras* K. Schum.	Tiliaceae	Leba Giraf	S	Ringworm	R	F/D	2	O		886
				Dermatophilosis	R	F	2	O		
*Vernonia amygdalina* Del.	Asteraceae	Girawa	T	Retained placenta/fetal membrane	R	F	5	O		22
				CBPP	L	F	5	O		
*Withania somnifera* (L.) Dun.	Solanaceae	Gizewa	S	Blackleg	R	F/D	5	O		206
*Zingiber officinale* Roscoe	Zingiberaceae	Zingibil	H	Bloat	Rh	F/D	5	O		
				Bloody diarrhoea	Rh	F/D	5	O	22	850
				Poor appetite	Rh	F/D	5	O		
				Mucal diarrhoea	Rh	F/D	5	O		

Key: Growth form Tree (T); Shrub (S); Herb (H); Climber (C). Part used (Leaf, L; Root, R; Stem wood, St; Fruit, Fr; Bark, B; Root bark, RB; Stem bark, SB; Flower, Fl; Bulb, Bu; Rhizome, Rh; Latex, Lat). Conditions of part used (CPU) (Dry, D; Fresh, F). Methods of preparation and application (MPAP), 1. Boil, keep to cool for some time and let the animal drink the decoction; 2. Grind and cover surface of diseased parts with the powder; 3. Grind and paste the crushed part 4. Extract the juice/oil/latex and pour or paint it; 5. Pound, homogenize with cold water and allow the animal to drink it or pour it on infected part. Route of Administration; (RA) (Oral, O; Dermal, De; Nasal, Na; Optical, Op).

**Endemic species.

Regarding the growth forms of plants of ethnoveterinary importance, there were more shrubs (23 species, 45%) followed by herbs (13, 25%), trees (10, 20%) and climbers (5, 10%). About 80% (41 species) of medicinal plants were those that are harvested from the wild environment, whereas 6% (3 species) from cultivation and the remaining 14% (7 species) were reported to be collected from both wild and cultivated sources. Deforestation (reported by 89% of informants), agricultural expansion (80%), charcoal making and firewood collection (33% collectively) and overgrazing (29%) were claimed as major factors affecting the medicinal plant wealth of the area.

### Ethnoveterinary plant knowledge of the people

The average number of medicinal plants reported by male informants (4.23±0.13) was found to be higher than that of female respondents (3.85±0.19), although the difference was statistically non-significant (P>0.05). Older informants (40–80 years old) who are senior members of the community reported significantly (P<0.05) higher numbers of medicinal plants than young to middle aged members. Similarly, there was a significant difference (P<0.05) in the number of medicinal plants reported by key informants and general respondents. Illiterate members of the community knew significantly more medicinal plants than literate ones (Table 2).

**Table 2:** Statistical test of significance on average number of reported medicinal plants among different informant groups in Ankober District

**Parameters**	**Informant groups**	**N**	**Average ± SD**	t -value**	**p –value**
Gender	Male	235	4.23 ± 0.13	1.61	0.1075
	Female	117	3.85 ±0.19		
Age	Young members	122	2.59 ±0.08	-11.65	0.0001*
	Senior members	230	4.90 ±0.13		
Literacy level	Illiterate	85	4.73 ±0.11	-11.90	0.0001*
	Literate	267	2.12 ± 0.10		
Experience (Informant category)	Key/knowledgeable	88	6.94 ±0.16	23.88	0.0001*
	General informant	264	3.16 ±0.07		

*Significant difference (p<0.05); **t(0.05) (two tailed), df = 350; N= Number of respondents.

### Types of livestock ailments and traditional diagnosis methods

A total of 33 veterinary ailment types were identified in the study area for which informants reported to use one or more medicinal plant species to treat specific livestock ailment type (Table 1). A majority of (18%, 6 ailment types) veterinary ailments claimed by the community belong to gastro-intestinal disease categories, followed by dermatological (15%, 5), and parasitic and respiratory (12% each, 4) disease categories. Diarrhoea and Mange were found to be the most commonly reported forms of veterinary ailments in the District.

Traditional medicine practitioners diagnose veterinary ailments based on observation and/or information obtained by interviewing the livestock owner about major symptoms shown by the diseased animal. Ethnoveterinary herbal remedies are commonly prescribed only after the diseased animal is visually examined by a traditional healer for any symptom on its mouth and foot parts, throat, eyes, nose, ear and/or skin as well as through presence and status of sores/wounds or any form of infection. Rubbing and pasting ethnoveterinary herbal preparations were the commonly reported treatment methods for handling dermatological diseases, whereas oral administration of homogenized herbal preparations was reported as the main treatment method for internal ailments.

### Applications of ethnoveterinary remedies

**Figure 2:**
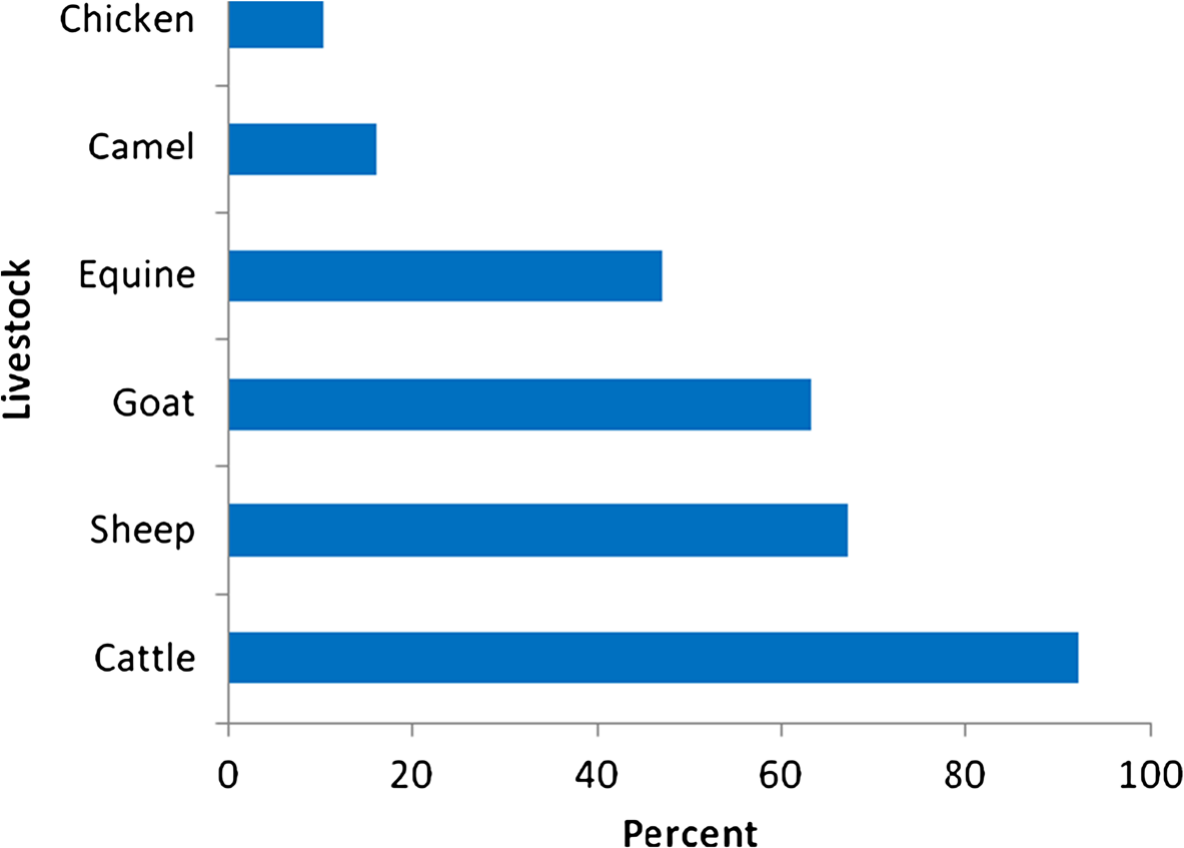
Percentage of ethnoveterinary medicinal plants used for different livestock types in Ankober District.

Although ethnoveterinary medicinal plants of the District are claimed to be applied for ailments affecting cattle, sheep, goats, equines, camels or chicken, the majority of the reported medicinal plants (92%, 47 species) were found to be applied to treat one or more of the thirty different cattle ailments (Table 1). A relatively few number of ethnomedicinal species (10%, 5 species) was mentioned to be used against chicken ailments (Figure 2).

Most ethnoveterinary medications (86%) were reported to comprise remedial parts of a single medicinal plant. However, 14% were prepared using formulations from two or more medicinal plant species. Amongst all plants reported, the highest proportion of species was claimed to treat diarrhoea (24%), mange (20%), ringworm and black leg (16% each) and bloat (14%). The highest number of multiple ethnoveterinary uses were recorded for *Allium sativum* (used against 8 ailment types) and *Croton macrostachyus* (7 ailment types) (Table 1).

### Plant parts used for remedy preparation

**Figure 3:**
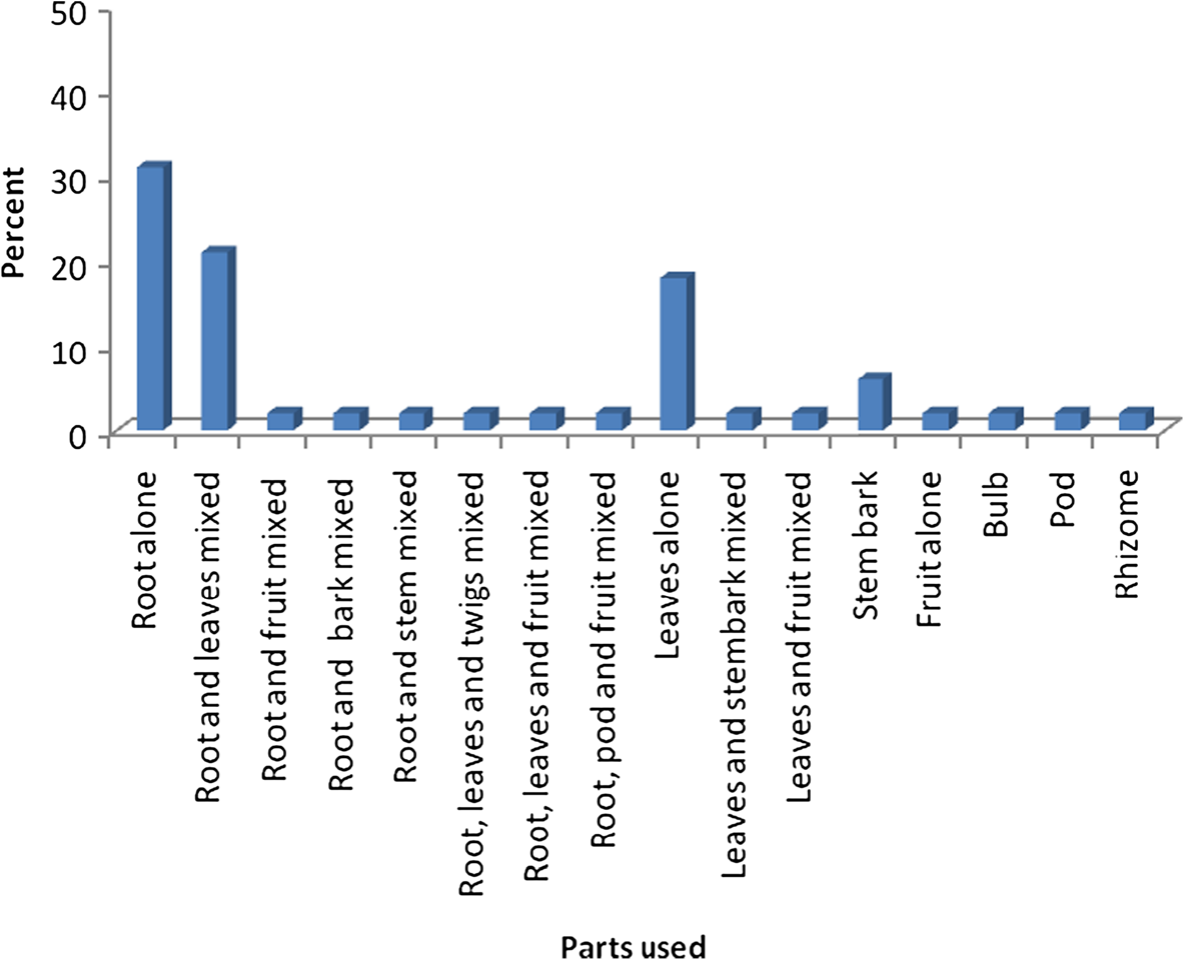
Plant parts used for remedy preparation in Ankober District.

Although different plant parts are reported to be used for remedy preparation by the community, a majority (31%) of preparations was found to be from root parts alone, followed by mixtures of leaves and roots (21%) and leaves alone (18%) (Figure 3). Plants in which roots (65%, 33 species) and leaves (43%, 22 species) are utilized as ingredient either alone or mixed with other plant parts were frequent in the medicinal flora of the District. Freshly harvested plant parts were the dominant ones (83%) used in remedy preparation whereas the remaining 17% of remedies were reported to be prepared both from dried or fresh parts of medicinal plant species.

### Modes of remedy preparation, routes of administration and dosages

**Figure 4:**
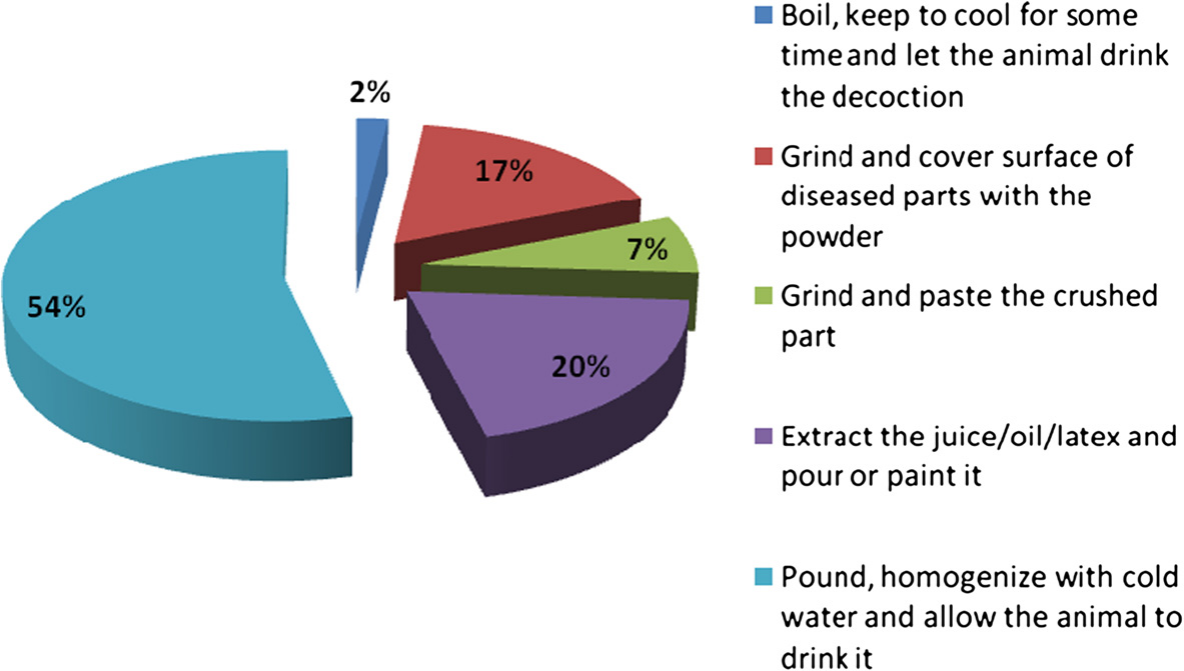
Modes of remedy preparation for treating livestock ailments in Ankober District.

Various modes of remedy preparation were reported to be used in the District based on type and degree of complexity of livestock ailment. Pounding the remedial part and homogenizing it with cold water was found to be the major mode of remedy preparation (54%), followed by extracting juice, oil or latex from the plant (20%) (Figure 4).

Traditional plant remedies were reported to be administered through oral, dermal, nasal or optical routes of the diseased animal. Oral application was the best-represented route of administration (69 preparations, 53%), followed by dermal (53, 40%), nasal (6, 5%) and optical (3, 2%) routes.

Physical appearance of the diseased animal and visually confirmed degree of complexity of illness were used to determine preparation doses to treat livestock ailments. Some traditional practitioners reported use of plastic jugs, glasses, bottles, cups or spoons to determine dosage for some medicinal preparations while others reported to use a handful or finger-sized preparation to treat ailments. However, no strictly standardised doses of herbal preparations as known for modern veterinary medicine were reported by traditional healers for any of the preparations used to treat livestock ailments in Ankober District.

### Marketability of medicinal plants of ethnoveterinary importance

About 20% (10 species) of ethnoveterinary plants were reported to be marketable and could be accessed through purchase at Aliuamba, Derefo, Gorebella, Gorgo, Haramba and Zego local markets in the District. However, out of ten reportedly marketable species only 2, i.e., *Embelia schimperi* Vatke and *Withania somnifera* (L.) Dun seem to be purchased utterly for the purpose of their traditional medical applications according to customer explanations. The remaining eight plants mentioned are mainly sold for their non-medicinal uses, but also applied as medicine when the need arises. This means that these eight species are frequently used for non-veterinary purposes but infrequently used for veterinary purposes. The average price of a bunch (**≈**250-325 g) of the root material of *Withania somnifera* at five local markets of the District was 3.50 Birr (0.2 USD), whereas the price for a cupful of the fruits of *Embelia schimperi* was 5.50 Eth Birr (0.3 USD).

### Test of efficacy of ethnoveterinary medicinal plants

Nine major livestock ailment categories were identified from the total 33 veterinary diseases reported in the District. Highest Informants’ Consensus Factor (ICF) values were recorded for gastro-intestinal (0.71), ecto and endo-parasitic (0.69) and dermatological disease (0.66) categories (Table 3). In addition, highest plant use citation (25.95%) was recorded for dermatological diseases.

**Table 3:** ICF values of traditional medicinal plants for treating livestock ailments in Ankober

**No**	**Disease category**	**Species**	**% all species**	**Use citations**	**% all use citations**	**ICF**
1	Gastro-intestinal	7	13.72	22	16.79	0.71
2	Ecto and endo parasitic	5	9.8	14	10.68	0.69
3	Dermatological	12	23.5	34	25.95	0.66
4	Sensorial	3	5.88	5	3.81	0.50
5	Respiratory	7	13.72	11	8.39	0.40
6	External injuries, bleeding and poisoning	11	21.56	18	13.74	0.40
7	Reproductive	3	5.88	4	3.05	0.30
8	Musculo-skeletal	8	15.68	9	6.87	0.13
9	Others	12	23.50	14	10.68	0.20

### Relative healing potential of medicinal plants

*Embelia schimperi* Vatke showed highest fidelity level value (90%) for gastrointestinal disease category followed by *Rubus steudnerii* Schweinf. (84%). Under the dermatological therapeutic category, highest fidelity level value was recorded for *Croton macrostachyus* Del. (83%). *Achyranthes aspera* L. (81%) has also showed relatively high healing potential record under the external injuries, bleeding and poisoning disease category (Table 4).

**Table 4:** Fidelity level value of medicinal plants commonly reported against a given veterinary ailment category

**No**	**Medicinal plant**	**Therapeutic category**	**IP**	**IU**	**FL Value%**
1	*Embelia schimperi* Vatke	Gastro-intestinal	36	40	90
2	*Rubus steudnerii* Schweinf.	Gastro-intestinal	26	31	84
3	*Croton macrostachyus* Del.	Dermatological	25	30	83
4	*Achyranthes aspera* L.	External injuries, bleeding and poisoning	39	48	81
5	*Phytolacca dodecandra* L’ Herit.	Ecto and endo-parasites	23	29	79
6	*Cissampelos mucronata* A. Rich.	Respiratory	24	32	75
7	*Trichocladus ellipticus* Eckl. & Zeyh.	Sensorial	19	27	70
8	*Withania somnifera* (L.) Dun.	Musculo-skeletal	28	39	71
9	*Aeonium leucoblepharum* Webb ex A. Richard	Reproductive	17	23	74
10	*Ocimum lamiifoium* Hochst.	Gastro-intestinal	30	38	66

### Preference ranking of ethnoveterinary plants

Preference ranking exercise with 15 of the key informants (selected randomly) for medicinal plants that were reported to be used against diarrhoea, the most frequently reported livestock disease under the gastro-intestinal disease category, showed that *Embelia schimperi* Vatke and *Rubus steudnerii* Schweinf. were most-preferred species to treat the reported disease (Table 5).

**Table 5:** Results of preference ranking of medicinal plants reported for treating livestock Diarrhoea

**Medicinal plants for diarrhoea**	**Informants labelled A to O**																
	**A**	**B**	**C**	**D**	**E**	**F**	**G**	**H**	**I**	**J**	**K**	**L**	**M**	**N**	**O**	**Total score**	**Rank**
*Dodonea angustifolia* L. f.	1	3	3	2	2	3	1	2	3	4	2	3	4	7	7	47	**5**
*Gomphocarpus fruticosus* (L.) Ait. F.	2	1	2	3	1	1	5	3	1	3	1	4	2	3	2	34	**6**
*Zingiber officinale* Roscoe	6	7	6	5	5	6	4	4	5	7	5	7	6	4	4	81	**3**
*Clutia abyssinica* Jaub. & Spach	3	2	1	1	3	4	2	1	2	2	3	2	3	1	1	31	**7**
*Ocimum lamiifolium* Hochst.	4	4	4	6	4	2	3	6	7	1	4	1	1	2	3	52	**4**
*Rubus steudnerii* Schweinf.	5	6	5	7	6	5	7	5	4	5	6	5	5	5	6	82	**2**
*Embelia schimperi* Vatke	7	5	7	4	7	7	6	7	6	6	7	6	7	6	5	93	**1**

**N:B**-Scores in the table indicate ranks given to medicinal plants based on their efficacy. Highest number (7) for the medicinal plant which informants thought was most effective in treating diarrhoea and the lowest number (1) for the least-effective plant.

## Discussion and conclusion

### Indigenous use and diversity of ethnoveterinary plants

The indigenous people of Ankober District rely on livestock as a major support to their livelihoods, employment, crop production, transport and for generating revenue to sustain life. Understanding indigenous knowledge, attitudes and practices of traditional communities about occurrence, treatment, prevention, control and local importance of different livestock ailments and traditionally used medicinal plants against respective ailments is crucial to design and implement meaningful animal health improvement and production strategies[37]. According to Pieroni and co investigators [16], ethnoveterinary studies have long-term output of developing eco-sustainable projects with a primary goal of using plant-based remedies in traditional and also new agricultural and animal breeding systems. Results of this investigation show that people in the District have age-old indigenous knowledge on the use of plants in the wild to treat various livestock ailments. The deep-rooted culture of plant use for successive generations might have played the role for a sentimental adherence of the community to ancestral medical traditions which are still held as highly valued heritage of the society.

One of the driving factors which made the people of Ankober District to rely on wild plants of ethnoveterinary importance to treat veterinary ailments is inadequate number of formal veterinary clinics (only five) and veterinarians (only eight) available in the area that would never be enough to provide healthcare services for more than 200,000 livestock population [46]. Moreover, almost all of the rural community lives in marginal areas which are not easily accessible to the rare modern veterinary services which are also known for their scorching prices totally unaffordable to the less economically endowed people living there.

Identified ethnoveterinary medicinal flora of Ankober showed that the District is rich in its ethnoveterinary plant diversity and indigenous knowledge associated with each traditionally used species. Comparison of the number and diversity of ethnoveterinary medicinal plant species used in Ankober with other ethnoveterinary research results of cultural communities in Ethiopia [7,40,42,43,63] and elsewhere in Uganda [64], Kenya [15], South Africa [65], Pakistan [66] and India [67,68] confirms the richness of the area in diversity of ethnoveterinary plants. The millennia-old interaction of indigenous people in the area with locally available medicinal plants might have enabled them to develop an indigenous knowledge system best fit to select and use diverse curative medicinal plants to treat frequently occurring livestock diseases. According to Rindos [69], knowledge on plant use is the result of many years of human interaction and selection on the most desirable and successful plants present in the immediate environment at a given time.

Some ethnoveterinary species of Ankober are also found with use reports in other ethno-linguistic communities in the country. Examples are *Calotropis procera* (Ait.) Aitf and *Withania somnifera* (L.) Dun. that also occur in the ethnoveterinary medicinal flora of the Afar people [43]; *Croton macrostachyus* Del., and *Ricinus communis* L. recorded for the Gilgel Ghibe area and Borana pastoralists [7,44]; and *Calpurnia aurea* (Ait.) Benth. and *Achyranthes aspera* L. reported for Tanqua-Abergele and Kolla-Tembien Districts [63]. Similarity of medicinal plant species used in different communities can be attributed to cross-fertilization of cultural knowledge among different ethno-linguistic groups besides distribution/availability of the species in use in areas investigated for their ethnomedicinal knowledge.

Best-representation of ethnoveterinary species from families Asteraceae, Asclepiadaceae, Euphorbiaceae and Ranunculaceae could be related to their wider distribution and use in Ethiopia/the flora area [55,56,58,61]. These families were also reported to have the largest share of ethnomedicinal species in other ethnobotanical inventories [42,43,63,70].

### Growth habit, source and remedial parts of Ethnoveterinary plants

Results also showed most-frequent utilization of shrubs followed by herbs in remedy preparation. Dominance of shrubs was also reported by earlier ethnobotanical inventories [30,71-73]. In contrast, other investigators [64,70,74-76] reported dominance of herbaceous species for ethnomedicinal preparation in Ethiopia and elsewhere. However, the variation in dominance of growth forms of medicinal plants used among different ethno-linguistic groups in the country could be attributed to the wide agro-ecological diversity and specific indigenous knowledge of different communities. Results also evidenced the dominant practice of harvesting majority (80%) of ethnoveterinary plants of Ankober from non-cultivated sources. This would indicate the extent of anthropogenic pressure exerted on wild plant resources of the area. Overdependence on wild resources coupled with shrinking of the wild habitat due to ever-increasing population pressure pose a threat to medicinal plant wealth of the area. Comparable trends in overharvesting medicinal plants from the wild were also reported [30,44,64,77-79].

The finding of roots to be the most harvested plant parts used for ethnoveterinary remedy preparation in the District might be associated with traditional beliefs in different communities about a powerful therapeutic effect of root parts for treating various ailments [80-82]. However, harvesting roots for remedy preparation is always accompanied with complete removal of the respective medicinal plant from the natural environment has been observed in many cases posing challenges by affecting eventual survival of the individual and ultimately the species. According to Sheldon *et al*. [83], the main factor to be considered for conservation and sustainable use of medicinal plants is the particular plant part harvested for its curative value and the way it is harvested. Although leaves were also reported to be harvested from many ethnoveterinary plants in Ankober, their harvesting could be regarded as sustainable since some leaves are left on the parent plant to carry on its life activities. Overharvesting of roots for remedy preparation was also reported for different cultural groups in the country and elsewhere [30,31,64,70,78,84] calling for a coordinated conservation measure built on local sustainable harvesting methods like not removing the major roots and covering the expose parts to save the fast eroding therapeutic plants ruthlessly hunted for their roots. Results also indicate pronounced preference of traditional healers in Ankober to make use of freshly harvested plant parts (83%) for remedy preparation over dried forms. This could be attributed to the wide-spread traditional belief of attaining high efficacy from fresh remedies due to higher presence of active ingredients in the form of secondary metabolites in the cases of fresh plant parts which community members rightly thought could be lost on drying. Similar observations were reported [30,70] for other cultural groups living in Ethiopia.

### Ethnoveterinary plant knowledge of the community

Comparison of medicinal plant knowledge held among community members of varying age groups in Ankober showed a significant difference (P<0.05) in plant use by senior/elderly members of the community (4.90±0.13) over younger ones (2.59±0.08). This could be related to a higher degree of cultural contact and experience of the elderly members with curative plants than that of younger members in the community as described by Silva and co-workers [85]. Modernization and high degree of secrecy on passing knowledge on medicinal plants within the family circle only to elder sons, and lack of interest on traditional remedies by younger groups might also explain the decline of indigenous knowledge going down the generation ladder in the District. Ethnobotanical inventories in Ethiopia [30,40,79,84,86] and elsewhere in other countries [85,87,88] also share a similar concern on the knowledge gap down generations in different cultural groups. Systematic documentation of indigenous knowledge on medicinal plant use through ethnobotanical inventories is essential to safeguard such fast-eroding knowledge among successive generations. The difference in knowledge gap observed among key and general informants in this investigation could also be associated with high degree of experience/specialisation and greater contact with therapeutic plants in the first than the latter group [89]. Results showed that both men and women members of the community in Ankober are knowledgeable on medicinal plant use despite the relative dominance of medicinal plant tradition by men which could be associated with the traditional flow of information along the male line in the country [84].

### Livestock ailments, remedy preparation and traditional diagnosis methods

The observed frequent utilization of finely pounded remedial parts which are then homogenized with cold water (54%) and used against various ailments could relate to age- old traditional experiences on proven efficacy of such products. Homogenizing remedial parts with water has also been reported from other cultural groups [30,70,80] against various ailments. The observed trend of using two or more medicinal plants (accounting for about 14% of preparations) for treating livestock ailments may be attributed to the expected synergistic effect of combinations of parts and their bioactive ingredients to treat ailments. Giday *et al*. [90] have also reported therapeutic efficacy of combinations of medicinal plant parts used in Shinasha, Agew-awi and Amhara peoples living in northwest Ethiopia for treating various ailments.

Identification of specific livestock ailment types in the area was found to be made based on age-old cultural knowledge on symptoms and corresponding livestock illnesses held in the memories of indigenous people. The same was found true in selecting curative plants which were thought to be most appropriate to heal different veterinary diseases. However, no standardised volume or weight measurements were set by traditional healers on the amount of herbal prescriptions for different livestock ailments. Ethnoveterinary studies conducted in Pakistan [91] and Brazil [20] have also reported the lack of standardised doses in traditional prescriptions of livestock remedies. In addition, similar patterns of diagnosis and herbal prescriptions were reported [30,70,71,73-75]. According to Abebe [92], lack of precision and standardization has been cited as one of the most important shortcomings of the traditional healthcare system in Ethiopia.

The observed diverse medicinal uses of plants in the area against various livestock ailments indicates presence of inherent curative property engraved within each medicinal plant which still plays an essential role for the production of relatively healthier cattle, sheep, goats, equines, camels or chicken. The relatively high number of medicinal plants cited for treating cattle ailments may also be related to incidence of more diseases affecting cattle populations in the area. Similarly, the highest proportion of medicinal plants used to treat dermatological diseases in the study area could also be related to high incidence of such diseases in Ankober. This was justified by presence of highest number of dermatological diseases (accounting 15% of livestock ailments in Ankober District) reported by community members. In addition, the finding indicates that traditional veterinary practices in the area are well focussed to the most prevalent health problems. Presence of largest share of ethnoveterinary plants for treating cattle ailments was also reported for other cultural groups in the country [39].

### Marketability of ethnoveterinary plants

Local communities in Ankober mainly collect and use ethnoveterinary plants for a private use with their own livestock. However, a limited scale of buying and selling traditional medicine was observed at Aliuamba, Derefo, Gorebella, Gorgo, Haramba and Zego local markets in the District. Though 20% (10 species) of ethnoveterinary medicinal plants were shown to be available on the market only 2 species i.e., *Embelia schimperi* (0.3 USD per cup of fruits) and *Withania somnifera* (0.2 USD per bunch of root material) seem to be purchased solely for use in traditional medical applications. Market price of these medicinal plants mainly depends on the distance travelled to collect remedial plants and availability of species in the area. *Embelia schimperi* and *Withania somnifera* were reported for scarcity in the area by informants and local retailers whereby the latter was explained to be due to overharvesting of species. This made collectors travel long distances to gather these species*.* The remaining eight marketed species were mainly sold for their uses other than medicine i.e., food, lumbering and firewood purposes though they were mentioned to be occasionally used as remedies when there is a need. Local retailers of medicinal plants, though not herbalists, have also shown some knowledge on use, local distribution and local market demand of economically useful medicinal plants which in turn indicated that knowledge on curative plants is not limited only to local healers or traditional herbalists. Status and availability of marketable medicinal plants were also reported in various sources [93-96] for other cultural groups in Ethiopia.

### Test of efficacy, healing potential and ranking of ethnoveterinary plants

The observed high informants’ consensus (ICF=0.71) on ethnoveterinary medicinal plants used to treat gastro-intestinal diseases in the area indicated popularity of curative plants against diseases in the latter category. Sharma *et al*. [22] have explained that high ICF values indicate highest share of similar plant use information within a community. The recorded high plant use citation (25.95%) for treating ailments in the dermatological disease category may also indicate the relatively high incidence of such diseases and ease of identifying ailments and corresponding curative plants occurring in the District. Since high ICF values are indicative of selecting target plants for search of bioactive compounds [62], our research team has used the results obtained in this investigation to select 21 elite species for further investigation of their pharmacological properties.

Highest fidelity level values were obtained for *Embelia schimperi* (90%) and *Rubus steudnerii* (84%) in the gastro-intestinal disease category, and *Croton macrostachyus* (83%) for the dermatological therapeutic category indicates relatively high healing potential of the species for treating ailments under the respective ailment categories [62,97]. Thus, these results would call for pharmacological investigations on these plants since high percentage of informants agreed on their curative values. Hence, plants with high fidelity level value obtained in this investigation are now being tested for pharmacological activities by our research team.

Output of the preference ranking exercise also indicated that *Embelia schimperi* and *Rubus steudnerii* are the most-preferred ethnoveterinary medicinal plants used to treat diarrhoea, the most commonly reported disease in the area. This may be attributed to the presence of bioactive compounds against causative agents of diarrhoea in these species. Hence, both species are also being investigated for further antimicrobial activities against different microbial strains by the same research group.

Generally, results of this investigation evidenced a rich ethnoveterinary medicinal plants and indigenous knowledge on their utilization in Ankober District. It is therefore worth engaging in the conservation of these plants which in turn calls for conserving their habitats. This action would save many more plants as well as medicinal plants for humans. Validating bioactivity of ethnoveterinary medicinal plants that are most agreed for their curative role by the community is highly recommended to come up with further scientific evidence which can be used to support the livestock healthcare system in the country and globally in the years ahead.

## Competing interest

The authors declare that they have no competing interests.

## Authors’ contributions

All authors have equal contribution and all authors have read and approved the final manuscript.
